# Arts engagement and mental health: a social psychological framework

**DOI:** 10.3389/fpubh.2026.1839011

**Published:** 2026-06-10

**Authors:** Weida Zhang, Wenzhang Li, Mengling Li

**Affiliations:** 1 Huaibei Normal University, Huaibei, China; 2 Jiangxi Normal University, Nanchang, China; 3 Central China Normal University, Wuhan, China

**Keywords:** arts engagement, mental health, psychosocial resources, public mental health, social psychology

## Abstract

Research on arts engagement and mental health has expanded rapidly in recent years, and a growing body of evidence suggests that participation in artistic and cultural activities is associated with improved well-being, reduced psychological distress, and stronger social connectedness. However, theoretical explanations remain insufficiently integrated. Existing studies identify a wide range of possible mechanisms, including emotional expression, stress reduction, social interaction, identity formation, and meaning-making, yet these processes are often discussed in parallel rather than synthesized within a coherent explanatory framework. In addition, the literature frequently conflates everyday arts engagement with community-based programmes and formal therapeutic interventions, making it difficult to determine what is distinctive about arts engagement as a mental health resource in ordinary social life. In response to these limitations, this article offers a conceptual review and develops a social psychological framework for understanding how arts engagement may influence mental health. It focuses primarily on everyday and community-facing forms of arts engagement in ordinary social life, while drawing selectively on therapeutic and programme-based literature to clarify conceptual boundaries and mechanisms. The article argues that arts engagement should be understood not merely as a leisure activity or cultural exposure, but as a socially organized practice through which individuals gain access to psychosocial resources. To advance this argument, it proposes a four-layer framework comprising socially scaffolded affect regulation, connectedness and belonging, social identity, collective meaning, and the social cure, and agency, recognition, and narrative repair. By integrating these processes within a single conceptual model, the article clarifies how arts engagement may influence mental health, under what conditions these effects are most likely to emerge, and why a social psychological perspective provides a valuable framework for understanding these associations.

## 1 Introduction

Mental health has become an increasingly urgent concern in contemporary public health, social policy, and community life. At the same time, arts engagement has attracted growing scholarly attention as a potentially important resource for supporting psychological well-being and reducing mental distress. What was once often treated as a peripheral topic within cultural policy has now developed into a substantial interdisciplinary field spanning psychology, psychiatry, public health, sociology, and arts-and-health research. This shift reflects a broader change in how the arts are understood: rather than being seen only as sources of recreation or cultural enrichment, they are increasingly examined as practices that may contribute to mental health in meaningful and measurable ways.

A growing body of empirical research supports this development. Longitudinal and epidemiological studies have shown that arts and cultural engagement is associated with lower risk of depression and better mental health outcomes across the life course. For example, Fancourt and Tymoszuk (1) found that cultural engagement appeared to be an independent risk-reducing factor for incident depression in older adults. In related work, Fancourt and Steptoe (2) showed that socioeconomic status explains only part of the association between cultural engagement and mental health, suggesting that the relationship cannot be reduced simply to advantage in income, education, or class position. Population-based studies have also linked arts engagement with stronger social connectedness and broader well-being outcomes. Using data from the HEartS Survey, Perkins et al. (3) found that arts engagement was widely perceived to support social connectedness, while Tymoszuk et al. (4) reported that higher levels of arts engagement were associated with greater well-being and lower intense social loneliness at the population level. More recently, Mak et al. (5) demonstrated that the relationship between arts engagement and mental health is likely to be bidirectional: better mental health can facilitate engagement, while engagement itself may also predict subsequent mental health benefits. Taken together, these findings indicate that arts engagement is meaningfully related to mental health, but they also suggest that the relationship is more dynamic and complex than a simple one-way causal pathway.

Despite this progress, the literature remains theoretically fragmented. One reason is that explanations of mechanism frequently move across biological, psychological, behavioral, and social levels of analysis without specifying how these processes relate to one another. In consequence, discussions of arts engagement and mental health often invoke stress reduction, emotional expression, distraction, social support, identity formation, and meaning-making simultaneously, but rarely integrate them into a coherent account. A second problem is conceptual ambiguity. The term arts engagement is often used broadly to refer to everyday cultural participation, community arts programmes, Arts on Prescription initiatives, and creative arts therapies. Yet these forms of engagement differ substantially in context, structure, facilitation, and purpose. Reviews of creative arts therapies and participatory arts-based programmes for children and young people underscore this heterogeneity and suggest that different forms of arts-based activity may operate through partially distinct processes (6, 7). As a result, the field has accumulated a large body of evidence without yet arriving at a sufficiently precise explanation of how arts engagement may affect mental health in ordinary social life.

A further limitation is that the specifically social psychological dimensions of this relationship have not been systematically synthesized. This matters because many of the most plausible pathways linking arts engagement to mental health are inherently social psychological in nature. Classic work on the stress-buffering role of social support has shown that supportive relationships can protect individuals from the harmful effects of stress (8). Research on belongingness has argued that the need to form and maintain meaningful social bonds is a fundamental human motivation with major consequences for emotional well-being (9). Self-determination theory likewise identifies relatedness, competence, and autonomy as basic psychological needs that support psychological functioning and well-being (10). Most directly, the social identity approach to health has demonstrated that psychologically meaningful group memberships are not merely features of social context but important psychological resources that provide meaning, support, purpose, and efficacy (11, 12). In addition, a systematic review and meta-analysis by Steffens et al. (13) found that interventions designed to build social identification improve health and well-being outcomes, reinforcing the importance of group-based psychosocial resources in mental health.

From this perspective, arts engagement is theoretically important because it may be especially well suited to generate such resources. Participatory arts activities are not simply occasions for individual expression; they are also contexts in which people encounter others, share attention, create jointly meaningful experiences, and sometimes develop a sense of commonality or collective identity. Evidence from the HEartS Survey is particularly suggestive in this respect. Perkins et al. (3) found that arts engagement supported social connectedness through pathways such as social opportunities, sharing, commonality and belonging, and collective understanding. These findings indicate that arts participation may contribute to mental health not only because it is pleasurable or absorbing, but because it organizes forms of shared experience that can be converted into psychosocial resources. This proposition is also supported by research on arts-based groups in mental health settings. Williams et al. (14) found that identification with arts-based groups predicted improvements in mental well-being among adults with chronic mental health conditions, while Williams et al. (15) showed that participation in choir and creative writing groups supported recovery through psychosocial processes that aligned closely with the social cure framework. Likewise, a systematic review by Williams et al. (16) concluded that group singing may function as a promising social intervention for adults with mental health conditions, even though the evidence base remains methodologically uneven.

At the same time, a social psychological account must also explain what is distinctive about the arts. Not every group activity offers the same opportunities for embodiment, symbolism, affective expression, or public recognition. In a scoping review of mechanisms of change in the creative arts therapies, de Witte et al. (6) identified embodiment, concretization, and symbolism/metaphor as distinctive therapeutic processes. Similarly, Williams et al. (7), in their review of participatory arts-based programmes for children and young people, highlighted the role of play, communitas, non-coercive space, and embodied understanding in facilitating mental health benefits. These findings suggest that arts engagement may be especially consequential not simply because it creates social contact, but because it creates shared, symbolically mediated, and often embodied forms of participation through which difficult experiences can be expressed, externalized, and socially witnessed. However, available evidence also cautions against romanticized assumptions. Tymoszuk et al. (4) found that although greater arts engagement was associated with higher well-being and lower intense social loneliness, the most highly engaged participants also showed elevated odds of depression and intense emotional loneliness in some cases. Such findings indicate that the effects of arts engagement are not uniformly positive and that they are likely to vary according to mode of engagement, intensity of participation, setting, and wider social conditions.

Against this background, the present article aims to develop an integrated social psychological framework for understanding how arts engagement may influence mental health. The article is conceived as a conceptual and narrative review rather than an exhaustive systematic review, and its primary focus is on everyday and community-facing forms of arts engagement in ordinary social life. Evidence from Arts on Prescription, community programmes, and creative arts therapies is included selectively where it helps clarify mechanisms, boundaries, or points of contrast, but these contexts are not treated as interchangeable. Rather than treating arts engagement simply as a cultural exposure, leisure activity, or loosely defined well-being practice, the present article conceptualizes it as a socially organized form of participation through which individuals gain access to psychosocial resources. The discussion that follows therefore draws purposively on interdisciplinary research from arts engagement, mental health, social psychology, public health, and arts-and-health studies, with particular attention to recent longitudinal studies, systematic reviews, meta-analyses, and theory-relevant empirical work. Its purpose is theory integration and conceptual clarification rather than formal evidence grading or quantitative aggregation.

Specifically, the article has three objectives. First, it clarifies the conceptual boundaries of arts engagement in relation to adjacent constructs such as creative arts therapies and other arts-based interventions. Second, it synthesizes the key social psychological mechanisms that may account for the mental health effects of arts engagement. Third, it examines the conditions under which these mechanisms are most likely to operate, including differences in mode of engagement, social form, setting, and access to participation.

Recent reviews have advanced understanding of the mechanisms linking arts engagement and mental health, but they have generally aimed to map a broad range of biological, psychological, behavioral, and social pathways. The purpose of the present article is different. Rather than providing an exhaustive mechanisms review, this paper offers a focused social psychological account of how arts engagement may influence mental health in ordinary social life. Its contribution lies in integrating dispersed evidence into a four-layer framework centered on socially scaffolded affect regulation, connectedness and belonging, social identity, collective meaning, and the social cure, and agency, recognition, and narrative repair.

The significance of this article is both theoretical and practical. Theoretically, it addresses a clear gap in the existing literature by integrating dispersed findings into a coherent social psychological model. This is important because current accounts of mechanism are often broad but insufficiently organized, making it difficult to evaluate causal pathways, compare findings across populations, or specify why certain forms of arts engagement may be more beneficial than others. Practically, a clearer account of psychosocial action can inform the design of community arts programmes, arts-based mental health promotion, and future empirical studies focused on mediation, heterogeneity, and context. If arts engagement is understood as a source of socially generated psychological resources rather than merely as an individual leisure activity, then its role in mental health also becomes a question of access, participation, and social infrastructure. From a public health perspective, this framing positions arts engagement as part of psychosocial infrastructure whose effects depend on access, inclusion, and cultural provision.

## 2 A social psychological framework for understanding how arts engagement affects mental health

The central claim of this article is that arts engagement may influence mental health not merely because it offers enjoyment, distraction, or aesthetic stimulation, but because it functions as a socially organized practice through which psychosocial resources are generated, distributed, and experienced. This claim is consistent with the recent mechanisms literature, which has increasingly moved beyond simple exposure-based explanations and toward multilevel accounts that recognize affective, interpersonal, behavioral, and contextual processes as mutually constitutive rather than separable (5, 17). A social psychological perspective is especially useful here because it foregrounds how participation in artistic activity is embedded in relationships, identities, symbolic systems, and opportunities for recognition. In this view, the arts do not simply act on mental health; they shape the social and psychological conditions under which people regulate emotion, relate to others, derive meaning, and experience themselves as capable and valued social actors (12).

This framing also helps clarify why arts engagement should not be reduced to a single mechanism. The literature now suggests that its mental health effects are multiply determined and highly contextual. Longitudinal and panel evidence indicates that arts engagement is associated with lower mental distress, better mental health functioning, higher life satisfaction, and lower incident depression, but also that the relationship is bidirectional: mental health influences participation just as participation influences mental health (1, 5, 18). A mechanism-focused framework therefore needs to explain not only how arts engagement may produce benefit, but also what kinds of social and psychological resources it mobilizes, and why those resources may matter more in some contexts than in others.

**Figure 1:**
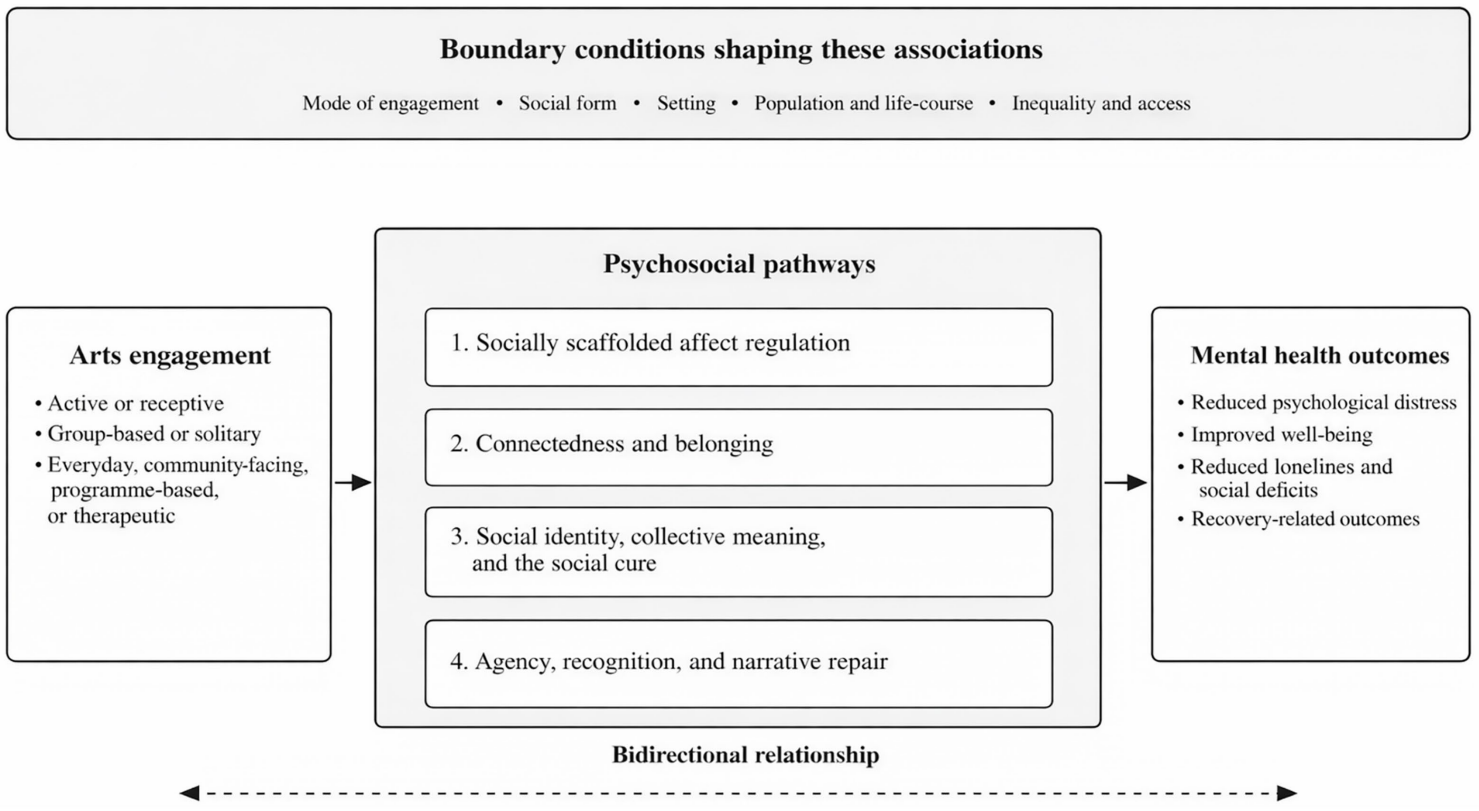
A social psychological framework linking arts engagement and mental health. Arts engagement may influence mental health through four interconnected psychosocial pathways: socially scaffolded affect regulation; connectedness and belonging; social identity, collective meaning, and the social cure; and agency, recognition, and narrative repair. These associations are shaped by boundary conditions including mode of engagement, social form, setting, population and life-course, and inequality and access. The relationship may also be bidirectional, insofar as mental health can influence the likelihood and form of subsequent engagement.

Building on this literature, the present article proposes a four-part social psychological framework. First, arts engagement can support socially scaffolded affect regulation, in which emotions are not managed solely within the individual but through structured, embodied, and symbolically mediated activity. Second, it can foster connectedness and belonging, creating opportunities for social contact to become subjectively meaningful social bonds. Third, it can promote social identity, collective meaning, and the social cure, transforming participation from simple co-presence into psychologically consequential group-based connection. Fourth, it can support agency, recognition, and narrative repair, enabling individuals to act, contribute, be seen, and rework valued versions of self. These processes are analytically distinct but often co-occur in practice. Importantly, the four pathways should not be understood as isolated or sequential mechanisms. In practice, arts engagement may first provide structured opportunities for affect regulation and social contact, which can then develop into belonging, shared identity, collective meaning, and experiences of agency and recognition. Thus, the framework is intended to show how psychosocial resources may accumulate and interact across affective, relational, identity-based, and agentic levels. Figure 1 summarizes the proposed framework and its principal boundary conditions.

### 2.1 Socially scaffolded affect regulation

Affect regulation is one of the most frequently cited mechanisms in arts-and-health research, but it is often described in ways that are overly individualistic. A social psychological account suggests that emotional regulation is rarely only an internal cognitive process. Rather, it is typically scaffolded by environments, symbols, social norms, and forms of activity that shape what can be felt, expressed, and managed. This is consistent with classic work on stress and social support, which showed that supportive contexts can buffer the effects of stress (8), and with self-determination theory, which emphasizes that psychological functioning depends partly on contexts that support basic needs for competence, autonomy, and relatedness (10). Arts engagement may be one such context: not merely a pleasant pastime, but a structured mode of participation through which affective experience becomes more manageable.

Recent mechanisms reviews reinforce this point. Fancourt et al. (17) argue that the mental health effects of arts engagement arise from interacting processes rather than isolated pathways, and they highlight emotional processing, attentional modulation, meaning-making, and interpersonal experience as interdependent rather than discrete. Likewise, de Witte et al. (6), in their scoping review of mechanisms of change in the creative arts therapies, identify embodiment, concretization, and symbolism/metaphor as especially characteristic processes. These mechanisms are important because they suggest that the arts do not simply permit emotional expression; they transform emotional material into forms that can be perceived, contained, revisited, and shared. What is otherwise diffuse, pre-verbal, or overwhelming can become structured through rhythm, image, narrative, gesture, or sound.

This is one reason why arts engagement may differ from many non-artistic leisure activities. The arts impose form on feeling. Rather than requiring direct disclosure, they often allow individuals to approach difficult experiences indirectly, aesthetically, and with a degree of symbolic distance. Perkins et al. (19), in a meta-ethnography of participatory music engagement, show that music can support mental well-being through emotional containment, expression, absorption, and reflection, while also creating a sense of safety through shared practice. Williams et al. (7), in their systematic review of participatory arts-based programmes for children and young people, similarly identify play, non-coercive space, embodied understanding, and communitas as key change processes. Together, these findings suggest that arts engagement supports affect regulation not through simple catharsis, but by creating environments in which emotion is both permissible and organized.

The affective value of arts engagement is also not limited to active participation. Recent evidence suggests that receptive engagement may matter as well, although potentially through different pathways. Trupp et al. (20), in a systematic review of viewing art and well-being, conclude that receptive engagement can support positive affect, reflection, and meaning, even though the evidence base remains methodologically heterogeneous. This is important because it suggests that the arts’ regulatory capacity does not depend exclusively on making or performing; it can also arise through aesthetic attention, interpretive engagement, and emotionally resonant perception. However, active and receptive forms likely scaffold different combinations of regulation, absorption, expression, and sociality, which is why they should not be analytically collapsed.

Finally, longitudinal evidence suggests that these processes may have population-level implications. Wang et al. (18), using fixed-effects analyses of a nationally representative panel, found that more frequent arts participation and cultural attendance were associated with lower mental distress and higher life satisfaction, independent of a wide range of time-invariant confounders. While such studies do not directly observe affective mechanisms, they are consistent with the proposition that repeated engagement in aesthetically and socially structured activities can help stabilize emotional life over time. The theoretical implication is that arts engagement may affect mental health not simply because it changes what people feel in the moment, but because it changes the social and symbolic conditions under which feelings are processed.

### 2.2 Connectedness and belonging

A second major pathway is connectedness. Across social psychology, belonging has long been understood as a fundamental human need, and Baumeister and Leary (9) argued that the desire to form and maintain meaningful interpersonal bonds is central to psychological functioning. From this perspective, the mental health significance of arts engagement lies not only in its capacity to regulate internal states, but also in its capacity to create conditions in which social contact can become meaningful, supportive, and enduring. Arts participation is theoretically important here because it often organizes interaction around shared activity rather than explicit self-disclosure. People make, view, listen, sing, perform, rehearse, move, and create with or alongside others. This gives sociality a task, an object, and a symbolic medium.

The strongest direct evidence for this pathway comes from the HEartS Survey. Perkins et al. (3) found that arts engagement supported social connectedness through four principal pathways: social opportunities, sharing, commonality and belonging, and collective understanding. These pathways are conceptually significant because they show that the social benefits of arts engagement are not reducible to mere attendance or co-presence. Rather, the arts appear to transform encounters into experiences of mutual recognition and shared understanding. Social opportunities create occasions for contact; sharing makes experience jointly held; commonality and belonging make others feel like people like me; and collective understanding allows participants to feel understood without necessarily requiring direct confession or explanation.

This distinction between contact and connection is crucial. Social psychological research has long shown that being around others is not sufficient for belonging. What matters is whether interaction becomes psychologically meaningful. In arts contexts, the shared object or practice can mediate this transition. Participation does not have to begin with intimacy in order to produce intimacy. The activity itself can carry some of the interpersonal burden. This may be particularly important for individuals experiencing distress, social withdrawal, stigma, or diminished confidence, for whom conventional forms of social participation can feel demanding or threatening. Arts engagement may therefore reduce the threshold for connection by allowing people to enter social situations through doing rather than disclosing.

Recent longitudinal work strengthens this argument. Noguchi et al. (21), using data from the Japan Gerontological Evaluation Study, found that arts and cultural engagement predicted lower subsequent social deficits among older adults. Bone et al. (22), in a longitudinal cross-country comparison of older adults in Japan and England, similarly reported that arts and cultural group participation could support later life satisfaction and aspects of social support, although the magnitude and consistency of effects varied by country. Bone et al. (23), using propensity score matching in a US sample of older adults, also found associations between community arts group participation and several aspects of well-being. Collectively, these studies suggest that the relational consequences of arts engagement are not merely subjective impressions; they can be observed prospectively in real-world populations.

Population-level evidence also indicates that the relationship is not simple or uniformly positive. Tymoszuk et al. (4), using HEartS data, showed that higher arts engagement was associated with higher well-being and lower intense social loneliness, but that the most highly engaged participants did not uniformly fare better across all outcomes. This matters theoretically because it reminds us that belonging is not an automatic outcome of participation. Arts engagement creates opportunities for connection, but whether those opportunities become stable psychosocial resources depends on context, continuity, and the subjective meaning of participation. Belonging must therefore be treated as a contingent outcome rather than an inevitable effect.

### 2.3 Social identity, collective meaning, and the social cure

Belonging is not the same as social identity. A key insight from the social identity approach to health is that what matters for mental health is not only whether people have contact with others, but whether that contact becomes psychologically meaningful as a shared we (11, 12). Group memberships can function as health-relevant resources because they provide shared meaning, support, purpose, and efficacy. Haslam et al. (24) go further in arguing that group-based social connection is especially consequential because it allows people to experience relational ties as identity-relevant rather than merely interpersonal. In this sense, arts engagement may matter not simply because it places people together, but because it can organize participation into shared identities and collective meanings.

This is one of the most important ways in which a social psychological perspective sharpens the arts-and-health literature. Many accounts of arts participation emphasize social interaction or community in broad terms, but social identity theory suggests that these categories are too loose. What matters is whether participants come to experience the group, practice, or activity as something that expresses who we are. Arts-based groups are especially well positioned to generate such identification because they are often built around repeated participation, shared symbols, common goals, public performance, and collective creation. These features can transform social contact into group-based belonging, and belonging into group-based meaning.

Empirical studies support this interpretation. Williams et al. (14) found that identification with arts-based groups predicted improvements in mental well-being among adults with chronic mental health conditions. This finding is theoretically important because it shows that the effect is not explained by attendance alone. Psychological identification with the group matters. Williams et al. (15) extend this argument in their study of choir and creative writing groups, showing that recovery-oriented benefits were closely aligned with the social cure framework: participants described belonging, positive affect, support, self-change, and purpose. These are precisely the kinds of psychosocial resources that the social identity approach predicts will matter for health and well-being.

The broader social identity literature lends further support to this interpretation. Jetten et al. (12) synthesize evidence showing that group memberships influence health through mechanisms such as social support, meaning, behavioral regulation, and efficacy. Steffens et al. (13), in a systematic review and meta-analysis, show that interventions designed to build social identification improve health and well-being outcomes. These findings are not arts-specific, but they are highly relevant because they help explain why arts-based participation may have mental health consequences beyond enjoyment or distraction. When arts engagement becomes a site of shared identity, it may unlock precisely the group-based psychological resources that social identity theory identifies as protective.

This also helps explain why group-based arts participation frequently shows stronger or more consistent associations with mental health than isolated forms of participation. Group singing, for example, has been repeatedly associated with benefits for adults with mental health conditions (16), and recent meta-analytic evidence among older adults suggests that group arts interventions can reduce depression and anxiety (25). While not all such effects should be attributed solely to identity processes, they are consistent with the view that collective participation can generate shared meaning and emotional synchrony that are more difficult to produce in solitary contexts. The concept of the social cure therefore provides more than a metaphor; it offers a mechanism-based explanation for why some arts experiences may become psychologically durable and mentally health-promoting.

### 2.4 Agency, recognition, and narrative repair

A fourth pathway concerns agency. Mental health is not only shaped by whether people feel calm or connected; it is also shaped by whether they experience themselves as capable of acting, contributing, and being recognized. Depression, chronic anxiety, and other forms of distress are often accompanied not only by negative affect but by reduced self-efficacy, diminished autonomy, social invisibility, and a damaged sense of self. Self-determination theory is useful here because it identifies competence, autonomy, and relatedness as core psychological needs (10). Arts engagement may be uniquely relevant to all three. It can involve skill, choice, improvisation, contribution, and relational exchange, often within publicly visible or socially acknowledged forms.

This matters because arts engagement positions people not only as recipients of support but as producers of value. To make, interpret, perform, or co-create something is different from merely being occupied. It can restore an active sense of self. Williams et al. (15) found that arts-based groups supported recovery through processes that included self-change and purpose, while Williams et al. (7) emphasize the importance of non-coercive participation, embodied understanding, and exploratory space. These features matter because they allow individuals to participate as agents rather than simply as cases, clients, or patients. Agency in this sense is not abstract volition; it is enacted through practice.

Community-arts and recovery literature makes this point even more explicit. Peters et al. (26), in a realist review of community arts, identity, and recovery, argue that community-based arts activities can support identity change processes in serious mental illness under particular contextual conditions. Their review is especially relevant because it moves beyond generic well-being claims and focuses directly on how arts participation can enable people to shift from stigmatized or problem-saturated identities toward more valued, socially sustainable forms of self-understanding. This is closely related to what might be called narrative repair: the process through which individuals rework the meaning of their experiences and their place within social life.

Recognition is central to this process. The psychosocial significance of artistic participation lies not only in private expression but in the possibility of being seen, heard, and responded to in ways that exceed clinical or deficit-based categories. De Witte et al. (6) show that symbolization and concretization allow difficult experiences to become externalized and worked with Baxter et al. (27), in their study of barriers and enablers to community and cultural engagement among people with lived experience of mental health conditions, demonstrate that participation is shaped by whether people feel psychologically safe, socially welcome, and able to imagine themselves in those spaces. Fancourt et al. (28) likewise show that individuals with depression and anxiety report distinctive barriers to participatory arts engagement across capability, opportunity, and motivation domains. These studies suggest that agency and recognition are not merely internal traits; they are relationally and institutionally produced.

Recent public-health-oriented work has pushed this point further by framing arts engagement as a health behavior and an equity issue. Rodriguez et al. (29) argue that arts engagement should be understood as a potential means of addressing mental health inequities, precisely because it can support psychosocial resources that are unequally distributed. This is an important extension of the agency argument. If the arts provide opportunities for expression, contribution, and recognition, then unequal access to arts participation also implies unequal access to these forms of psychosocial resource. From a social psychological perspective, then, arts engagement may matter for mental health not only because it helps people feel better, but because it can help them participate in social life as recognizable, capable, and valued subjects.

## 3 Boundary conditions of the mental health effects of arts engagement

### 3.1 Mode of engagement: active versus receptive participation

One major boundary condition is whether arts engagement is active or receptive. Active forms of engagement, such as singing, dancing, acting, crafting, creative writing, or visual art-making, may be especially likely to support agency, social identity, and embodied affect regulation because they involve contribution and practice. Receptive forms, such as visiting museums, watching performances, or viewing art, may still support reflection, emotion regulation, and meaning-making, but often through different combinations of attention, interpretation, and symbolic resonance (17, 20).

The recent adolescent literature illustrates this distinction clearly. Hugh-Jones and Munford (30), in a large UK panel study, report that arts and cultural engagement is associated with improved adolescent mental health, but that active engagement appears to confer stronger benefits than some more passive forms. Hugh-Jones et al. (31), in their systematic review and assessment of causality, likewise conclude that the evidence is strongest where engagement is regular and participatory, although causal certainty remains moderate rather than definitive. These findings do not imply that receptive engagement is unimportant. Rather, they suggest that different modes may mobilize different mechanisms, and that participatory forms may be especially potent where belonging, identification, and competence are central.

This distinction is also evident in older-adult evidence. Liu et al. (32), in an umbrella review of participatory arts-based interventions for older adults without dementia, emphasize the importance of artistic participation as a distinct form of engagement rather than simply aesthetic exposure. Quinn et al. (25) likewise focus specifically on group arts interventions, finding beneficial effects on depression and anxiety. Taken together, these studies suggest that the social psychological mechanisms most central to this review, belonging, identity, agency, and recognition, are likely to be particularly activated in participatory forms, even though receptive forms may still matter for affect regulation and meaning.

### 3.2 Social form: group-based versus solitary participation

A second boundary condition concerns whether participation is group-based or solitary. This distinction is theoretically crucial because several of the mechanisms emphasized in this review, including connectedness, social identity, collective meaning, and recognition, are intrinsically relational. Solitary participation may still support absorption, self-expression, or reflective processing, but it is less likely to generate group-based psychosocial resources unless it is embedded in a broader social ecology of discussion, shared interpretation, or community affiliation.

Recent evidence directly supports this distinction. Kaya and Mathieu (33), using the UK Household Longitudinal Study, found that frequent group-based arts participation was positively associated with psychological well-being, whereas solitary arts activities were not significantly associated with the same outcome once key confounders were taken into account. This is a particularly important finding for a social psychological account, because it suggests that the social form of participation is not a peripheral detail but a substantive determinant of mental health associations. Group arts and solitary arts are not interchangeable variants of the same underlying exposure.

Meta-analytic and review evidence points in the same direction. Quinn et al. (25) find that group arts interventions reduce depression and anxiety among older adults, while Williams et al. (16) conclude that group singing may be a promising intervention for adults with mental health conditions. Williams et al. (15) similarly show that choir and creative writing groups support recovery through social cure mechanisms. None of these findings prove that group formats are always superior, but they do indicate that the social form of participation shapes which psychosocial resources are most likely to be activated. Group settings may be especially consequential when the pathway of interest is belonging or identification; solitary settings may be more relevant where introspection, symbolic processing, or private regulation is central.

### 3.3 Setting: everyday engagement, community arts, arts on prescription, and therapeutic contexts

A third boundary condition concerns setting. The literature often uses arts engagement as a broad umbrella, but the contexts in which people engage with the arts differ substantially in institutional structure, facilitation, expectation, and intended outcome. Everyday arts engagement, community arts programmes, Arts on Prescription, and creative arts therapies should not be treated as functionally equivalent. They may overlap in some mechanisms, but they differ in the conditions under which those mechanisms are activated and sustained.

This distinction is especially important for theoretical precision. De Witte et al. (6) focus on creative arts therapies, where professional facilitation, therapeutic intent, and clinical framing are central. Peters et al. (26) focus on community-based arts activity in recovery contexts, where identity change unfolds through community participation rather than formal therapy. Jensen et al. (34), in their systematic review and meta-analysis of Arts on Prescription, examine a referral-based model that sits between community participation and targeted psychosocial intervention. Liu et al. (32) focus on participatory arts-based interventions in older adults. These are not the same object of analysis. If studies from all four contexts are combined without conceptual differentiation, mechanisms become difficult to interpret.

The setting also shapes participation norms and identity possibilities. Arts on Prescription programmes may lower access barriers and offer structured support, but they may also frame participation in quasi-clinical terms. Community arts programmes may be especially well suited to producing recognition and identity change outside explicitly pathological categories. Therapeutic settings may provide stronger containment and facilitation for intense affective work. Everyday arts engagement may be more ecologically valid and sustainable, but less structured. Any mechanism-based account needs to acknowledge these differences, because the same nominal activity, such as painting or singing, may have different psychosocial meanings depending on whether it occurs in a clinic, a community center, a social-prescribing pathway, or ordinary daily life.

### 3.4 Population and life-course variation

A fourth boundary condition concerns population and life-course. Different groups encounter the arts under different developmental, social, and clinical conditions, and the same arts practice may not carry the same psychosocial significance across age groups or populations. The youth literature, for example, emphasizes play, exploration, non-coercive space, and peer-based belonging (7), whereas older-adult studies often foreground social support, loneliness, and opportunities for sustaining well-being in later life (23, 25). Recovery-oriented studies among adults with chronic mental health conditions emphasize stigma, damaged identities, and the need for group-based meaning and recognition (14, 15, 26).

Recent adolescent evidence also suggests that the causal strength of arts engagement may differ by developmental stage and participation form. Hugh-Jones and Munford (30); Hugh-Jones et al. (31) show that regular arts engagement may support adolescent mental health, but also indicate that the evidence base remains uneven and methodologically varied. Among older adults, by contrast, recent meta-analytic and umbrella-review evidence has become relatively dense, especially for group-based interventions and participatory activities (25, 32). This asymmetry in the evidence base itself is important: some populations are now supported by multiple synthesis studies, whereas others still rely heavily on smaller or more heterogeneous designs.

Population differences are not merely demographic details; they shape which mechanisms are plausible and how they operate. Belonging may be especially salient where peer relations or social isolation are central. Agency and recognition may matter most in populations marked by stigma or identity threat. Affect regulation may be salient across the life course, but the forms through which it is scaffolded may differ by age, embodiment, and context. Future research therefore needs to treat population not as a control variable, but as a substantive moderator.

### 3.5 Inequality, access, and cultural infrastructure

A final boundary condition is inequality. Arts engagement is not distributed evenly across populations, and access to participation is shaped by socioeconomic resources, educational opportunity, cultural capital, geography, disability, mental health status, and institutional provision. This matters because if arts engagement functions as a source of psychosocial resources, then unequal access to participation also implies unequal access to mental-health-relevant supports.

The evidence here is increasingly strong. Fancourt and Steptoe (2) show that socioeconomic status does not fully explain the association between cultural engagement and mental health, but this should not be read as evidence that inequality is unimportant. Rather, it suggests that arts engagement has effects above and beyond socioeconomic position while remaining socially patterned. Fancourt and Baxter (35) show that poor mental health itself can be associated with lower participation in some community cultural activities. Fancourt et al. (28) further demonstrate that people with depression and anxiety report distinctive barriers across capability, opportunity, and motivation domains. Baxter et al. (27) add qualitative depth to this picture, showing that people with lived experience of mental health conditions encounter social, psychological, and practical barriers to community and cultural engagement. Rodriguez et al. (29) sharpen the policy relevance of this issue by arguing that arts engagement should be framed as a health behavior with implications for mental health inequities.

These findings have important theoretical consequences. They suggest that arts engagement should not be conceptualized only as an individual preference or voluntary leisure choice. It is also a matter of cultural infrastructure. Who is able to participate, how participation is supported, and whether individuals feel welcome in arts spaces are not secondary implementation issues; they are part of the causal ecology of mental health effects. A social psychological account that ignores access and inequality risks overestimating the universality of benefits and underestimating how structural conditions shape psychosocial resource distribution.

## 4 Future research agenda

The following priorities are intended not as an exhaustive agenda for the entire field, but as a focused research programme for testing, refining, and comparing the social psychological framework proposed here.

### 4.1 Conceptual precision and typologies of arts engagement

A first priority is conceptual clarification. One of the major obstacles to synthesis remains the tendency to blur together everyday arts engagement, community arts, Arts on Prescription, and creative arts therapies. These categories overlap, but they are not interchangeable. Sonke et al. (36) argue that public-health research requires more inclusive yet more precise definitions of arts participation. This is an important advance, because definitional ambiguity weakens theory, search strategies, measurement, and evidence synthesis. Future work should therefore develop clearer typologies that distinguish at minimum active versus receptive participation, solitary versus group-based participation, everyday versus intervention-based engagement, and community versus therapeutic settings.

This conceptual work is not preliminary housekeeping; it is part of mechanism development. As Fancourt et al. (17) note, mechanisms cannot be adequately specified when the underlying exposure is poorly defined. Typologies should be designed to help identify which psychosocial resources are likely to be activated in which forms of participation. More precise conceptualization would also allow future systematic reviews to compare like with like rather than aggregating fundamentally different practices under one label.

### 4.2 Mechanism-focused longitudinal and mediation research

A second priority is to move beyond mechanism listing toward mechanism testing. The current literature often identifies plausible pathways, including emotion regulation, belonging, identity, and agency, but relatively few studies directly test whether changes in these constructs mediate subsequent changes in mental health. Mak et al. (5) have already shown that arts engagement and mental health are bidirectionally related, which makes simple association studies increasingly insufficient. Future research should therefore prioritize longitudinal mediation designs, cross-lagged models, and other approaches capable of clarifying temporal ordering.

This agenda should be closely aligned with the social psychological framework proposed in this review. If belonging matters, then studies should test whether arts engagement predicts later mental health through changes in connectedness. If social identity matters, then identification with arts-based groups should be modeled explicitly, as in the work of Williams et al. (14, 15). If agency matters, then measures of competence, autonomy, public recognition, or identity change should be incorporated prospectively. The most valuable next-generation studies will not merely show that the arts help; they will identify which psychosocial variables carry that effect.

### 4.3 Stronger comparative designs

A third priority is comparative research. The field now needs more studies that directly compare active and receptive participation, group-based and solitary participation, everyday engagement and structured intervention, and arts activities with non-arts social activities. Kaya and Mathieu (33) provide one important model by distinguishing group-based from solitary arts participation in longitudinal data. Trupp et al. (20) provide another by focusing specifically on receptive art viewing. Quinn et al. (25) and Jensen et al. (34) focus on specific intervention models and populations. What is still missing are more systematic head-to-head comparisons capable of showing not only that multiple forms of engagement matter, but how their psychosocial profiles differ.

Comparative designs are especially important for theory refinement. If active participation shows stronger associations with agency and identity while receptive participation shows stronger associations with reflection or affect regulation, this would strengthen mechanism-based explanation. Likewise, if arts-based groups outperform non-arts groups on belonging or narrative repair, this would clarify what is distinctive about arts participation as opposed to social participation more generally. Such designs are more demanding, but they are essential for moving from plausible interpretation to explanatory precision.

### 4.4 Measurement harmonization and outcome coherence

A fourth priority is measurement harmonization. Recent systematic reviews repeatedly note heterogeneity in the way arts engagement and mental health outcomes are measured (17, 31, 32). This heterogeneity makes it difficult to compare findings across studies, populations, and interventions. Mental health outcomes range from depression and anxiety to life satisfaction, loneliness, quality of life, flourishing, and recovery-oriented constructs. Arts exposure is also measured inconsistently, with studies variously emphasizing frequency, intensity, modality, setting, or self-defined participation.

Future research should therefore work toward greater coherence in both exposure and outcome measurement. This does not require reducing the field to a single metric, but it does require more explicit rationale for measurement choices and better alignment between theory and indicators. If a study claims to examine belonging, identity, or recognition, it should measure those constructs directly rather than infer them from global well-being changes. If a study claims to examine arts participation, it should specify the social form, modality, and context of engagement. The conceptual work proposed by Sonke et al. (36) is highly relevant here, because more precise definitions can support better instruments, clearer coding frameworks, and more cumulative synthesis.

### 4.5 Equity, access, and implementation as Core research questions

A fifth priority is to integrate equity and implementation into mainstream theory rather than relegating them to policy afterthoughts. Research on barriers and enablers has already shown that people with poor mental health may face distinctive obstacles to participation (27, 28). Rodriguez et al. (29) argue that arts engagement should be treated as a health behavior relevant to mental health inequities, and Jensen et al. (34) highlight implementation challenges in Arts on Prescription pathways. These are not merely issues of program delivery; they shape who gets access to psychosocial resources and whether proposed mechanisms can operate at all.

Future studies should therefore pay more attention to inclusion, reach, retention, and the social distribution of participation opportunities. Questions of who participates, who drops out, who never reaches the programme, and which spaces feel culturally or psychologically accessible should be treated as central empirical questions. If arts engagement is understood as part of mental health infrastructure, then implementation and equity are not external to theory; they are part of the causal system being theorized.

### 4.6 From arts as benefit to arts as psychosocial infrastructure

Finally, the field would benefit from a broader conceptual shift. Much of the literature still asks whether the arts are beneficial for mental health, as though the arts were an optional add-on to otherwise sufficient psychosocial systems. A more theoretically ambitious approach would treat arts engagement as part of psychosocial infrastructure: one means by which affect regulation, social connection, identity formation, and recognition are made available in everyday life. This shift would better align research with public mental health concerns, especially where loneliness, disconnection, stigma, and diminished agency are widespread.

Such a reframing has practical implications. It suggests that the value of the arts lies not only in individual-level outcomes, but in whether social systems provide reliable opportunities for meaningful participation. It also implies that the most important research questions are no longer only whether people benefit from the arts, but how arts-based opportunities are embedded in communities, how they become sustainable, and how they can be made equitably available. From a social psychological perspective, this is the logical next step in the field’s development.

### 4.7 Limitations of the present review

The present article has several limitations. First, it is a conceptual and narrative review rather than a systematic review, and it therefore does not aim to provide exhaustive coverage of all studies in the field. Second, the evidence base remains uneven across populations, settings, and art forms, with relatively stronger synthesis evidence in some areas, such as older adults and group-based interventions, than in others. Third, many of the mechanisms discussed here remain theoretically plausible rather than directly tested through prospective mediation designs. The proposed framework should therefore be understood as a theory-generating and theory-organizing model that requires further empirical evaluation.

## 5 Conclusion

This conceptual review has argued that the relationship between arts engagement and mental health is best understood through a social psychological lens. Rather than treating arts engagement simply as a leisure activity, cultural exposure, or supplementary intervention, the article has conceptualized it as a socially organized practice through which psychosocial resources are generated, accessed, and sustained. From this perspective, the mental health significance of arts engagement lies not only in what people do, but in the affective, relational, and symbolic conditions that artistic participation makes possible in ordinary social life.

To develop this argument, the article proposed a four-layer framework in which arts engagement may influence mental health through socially scaffolded affect regulation, connectedness and belonging, social identity, collective meaning, and the social cure, and agency, recognition, and narrative repair. Taken together, these processes suggest that the arts matter for mental health not merely because they are enjoyable, distracting, or expressive, but because they can create conditions in which people regulate emotion, experience belonging, participate in meaningful groups, and sustain valued senses of self. A social psychological perspective is particularly useful in this regard because it helps explain how artistic participation may be converted into psychosocial resources with consequences for well-being, distress, loneliness, recovery, and related dimensions of mental health.

At the same time, this article has emphasized that these effects are neither universal nor context-free. The mental health consequences of arts engagement are shaped by mode of participation, social form, setting, population and life-course, and inequality of access. They are also likely to depend on the quality of facilitation, especially in community, prescription-based, and therapeutic contexts where facilitators help structure safety, inclusion, expression, and shared meaning. For this reason, arts engagement should not be romanticized as inherently beneficial in all circumstances. Its value depends on how participation is structured, who is able to take part, and whether artistic spaces become socially and psychologically meaningful for those involved. The relationship is also likely to be dynamic and, in some cases, bidirectional, such that mental health may influence both the likelihood and the form of engagement, just as engagement may influence subsequent mental health.

The broader contribution of this article is therefore both theoretical and practical. Theoretically, it offers an integrated account of how arts engagement may influence mental health by locating its effects within core social psychological processes rather than treating mechanisms as an unstructured list of possible explanations. Practically, it suggests that the role of the arts in mental health should be understood not only at the level of individual benefit, but also at the level of social and cultural infrastructure. If arts engagement provides access to psychosocial resources that support mental health, then access to such participation is also a matter of public health relevance. In this sense, arts engagement matters for mental health not simply because it enriches experience, but because it helps organize the social and symbolic resources through which mental health is lived and maintained.
